# Applying the structural equation model approach to study the simultaneous relationship between women’s empowerment and mental disorder in Egypt

**DOI:** 10.1186/s12905-023-02863-6

**Published:** 2024-01-06

**Authors:** Hassan H.M. Zaky, Dina M. Armanious, Maria A. Kalliny

**Affiliations:** 1 Department of Psychology, School of Humanities and Social Sciences, The American University in Cairo, Cairo, Egypt; 2 Present Address: Department of Statistics, Faculty of Economics and Political Science, Cairo University, Cairo, Egypt; 3 Social Research Center, The American University in Cairo, Cairo, Egypt

**Keywords:** Empowerment, Psychiatric Illness, Latent variable modelling, Multivariate analysis

## Abstract

**Purpose:**

The main purpose of this paper is to examine whether women’s empowerment and mental disorder affect each other in a one-way or two-way simultaneous relationship. Accordingly, the study fills a gap in the literature since it is the first attempt to examine the simultaneous relationship between women’s empowerment and mental disorder in Egypt. To achieve this, the study aims to examine the most important dimensions of women’s empowerment and mental disorder that affect each other simultaneously, and the most important determinants affecting women’s empowerment and mental disorder.

**Design/methodology/approach:**

The study depends on the cross-sectional data from the “Survey of Young People in Egypt” implemented in 2014. Married women aged 14–35 are included in the analysis (N = 3052). Recursive and nonrecursive structural equation models are used to examine the simultaneous relationship between women’s empowerment and mental disorder using AMOS, which stands for Analysis of Moment Structures (Version 22).

**Results:**

Women’s education has a positive significant impact on women’s empowerment and mental health. Violence has a positive significant impact on mental disorder, while it has a negative impact on women’s empowerment. Sexual harassment has a negative impact on treatment with spouse dimension. Regarding the one-way relationship, the results show that the more empowered the woman, the better her mental health is. Considering the two-way simultaneous relationship, the findings show that there is a partial two-way simultaneous relationship.

**Conclusion:**

There is a relationship between women’s empowerment and mental health, indicating that they affect each other simultaneously. Awareness of the importance of psychological counselling and treatment for mental disorders in women is needed.

## Background

In recent years, women’s empowerment and mental disorder became an important aims of international development policies, and many donor agencies now include these topics in their development. This study aims to examine the relationship between women’s empowerment and mental disorder among youth women in Egypt. Poor global mental health, especially among young people, has gradually been recognized as a major public health issue [1, 2]. Accordingly, there is a declaration that said “there is no health without mental health” [3]. Therefore, the definition of health in the World Health Organization constitution reflected the importance of mental health by “not merely the absence of disease or infirmity”, but rather, “a state of complete physical, mental and social well-being” [4]. Mental health can be defined as “a state of well-being enabling individuals to realize their abilities, cope with the normal stresses of life, work productively and fruitfully, and make a contribution to their communities” [5]. The person becomes mentally disordered if he or she suffers from any defect in the definition of mental health.

Mandal [6] defines women’s empowerment as ‘’an active, multidimensional process which enables women to realize their full identity and powers in all spheres of life’’.

The significance of the study was highlighted in many points. Firstly, the study fills a gap in the literature in Egypt since it is the first attempt to examine the simultaneous relationship between women’s empowerment and mental disorder in Egypt. Secondly, the data depends on youth married women and two types of structural equation models, recursive and nonrecursive models, to achieve the study goals. Finally, the study constructs new indicators (dimensions) representing women’s empowerment and mental disorder depending on the “Survey of Young People in Egypt (SYPE 2014)”.

Kermode et al. [7] studied the relationship between mental health and women’s empowerment in a qualitative manner, unlike our research. His study undertook a qualitative study on 32 women in rural Maharashtra in India. The key findings of this qualitative study show that women viewed the determinants of mental health and illness as mainly cultural and socioeconomic factors. Second, mental health was commonly conceptualized as an absence of stress, and the most identified stressors were conflicts with husbands and mothers-in-law, domestic violence and poverty. Finally, women’s mental health and empowerment were inextricably linked. Heim and Schaal [8] examine whether gender affects mental stress in Rwanda and apply nonrecursive structural equation modelling (SEM). The results show that gender is a significant predictor of mental stress.

The literature studies show that women’s empowerment dimensions could be measured by personal autonomy, household decision-making, economic domestic consultation and freedom of movement [9, 10]. Baig et al. [11] showed that the three dimensions of women’s empowerment, which are self-esteem, the power of decision-making and freedom of mobility, have a statistically positive significant impact on rural development, while control over resources was found to be insignificant. It also has been suggested that a high level of education and job opportunities can play vital roles in empowering women.

Logistic regression models were used in previous studies to assess the determinants of women’s empowerment. Jeckoniah et al. [10] showed that women’s education, media exposure, marital status, age at first marriage, land ownership, access to credit and living in urban areas played important roles in raising the level of women’s empowerment. Most scholars have shown that education has a significant impact on women’s empowerment [12, 13]. Ibrahim and Asad [14] illustrated that there is a positive correlation between the quality of education and women’s economic empowerment. The literature states that wealth status has a significant impact on women’s empowerment. Voronca et al. [15] found that women’s empowerment has increased over time in Kenya and is associated with increased family wealth. Additionally, women who are empowered are less likely to be at risk of domestic violence [16]. Scholars have found that sexual harassment has a weaker relationship with women’s negative self-views. Keplinger et al. [17] determined that women feel better supported and empowered and are not ashamed to speak up about sexual harassment. Modrek and Liu [18] mentioned that female circumcision has a significant impact on women’s empowerment, as socioeconomic status is the main driver of girls’ circumcision risk.

Hser et al. [19] showed that a poor physical health condition contributed to a higher probability of mental disorder. Ohrnberger et al. [20] found that there is a marked relationship between mental health and physical health where there are significant direct and indirect effects for both forms of health, with indirect effects explaining 10% of the effect of past mental health on physical health and 8% of the effect of past physical health on mental health.

Brown and Ciciurkaite [21] analysed data from a cross-sectional community survey of 455 heterosexual couples in which at least one partner had a physical disability; they examined the associations between stigma and psychological distress for both partners. The findings reveal that personally experienced stigma and vicarious stigma experiences have significant effects on psychological distress, but only among women.

Uddin et al. [22] showed that wealth status affects mental health by illustrating that poor people have the highest odds of hypertension compared with privileged rich group members. Niemeyer et al. [23] showed that depressive symptoms were more prevalent for persons with a low educational level. Oram et al. [24] showed that there is a direct link between women’s experiences of domestic violence and heightened rates of depression and poor mental health.

From the previous review of literature, it can be concluded that women’s empowerment is measured through the decision-making index, freedom of movement and regular spousal communication index. On the other hand, previous studies have shown that women’s education, employment status, age at first marriage, land ownership, living in urban areas play important roles in raising the level of women’s empowerment. The literature also showed that violence, stress, poverty, etc., have significant effects on mental health. Kermode et al. [7] examines the relationship between women’s empowerment and mental health who concluded that women’s mental health and empowerment were inextricably linked using a qualitative approach.

- **Conceptual Framework for the objectives of the study**.

**Fig. 1:**
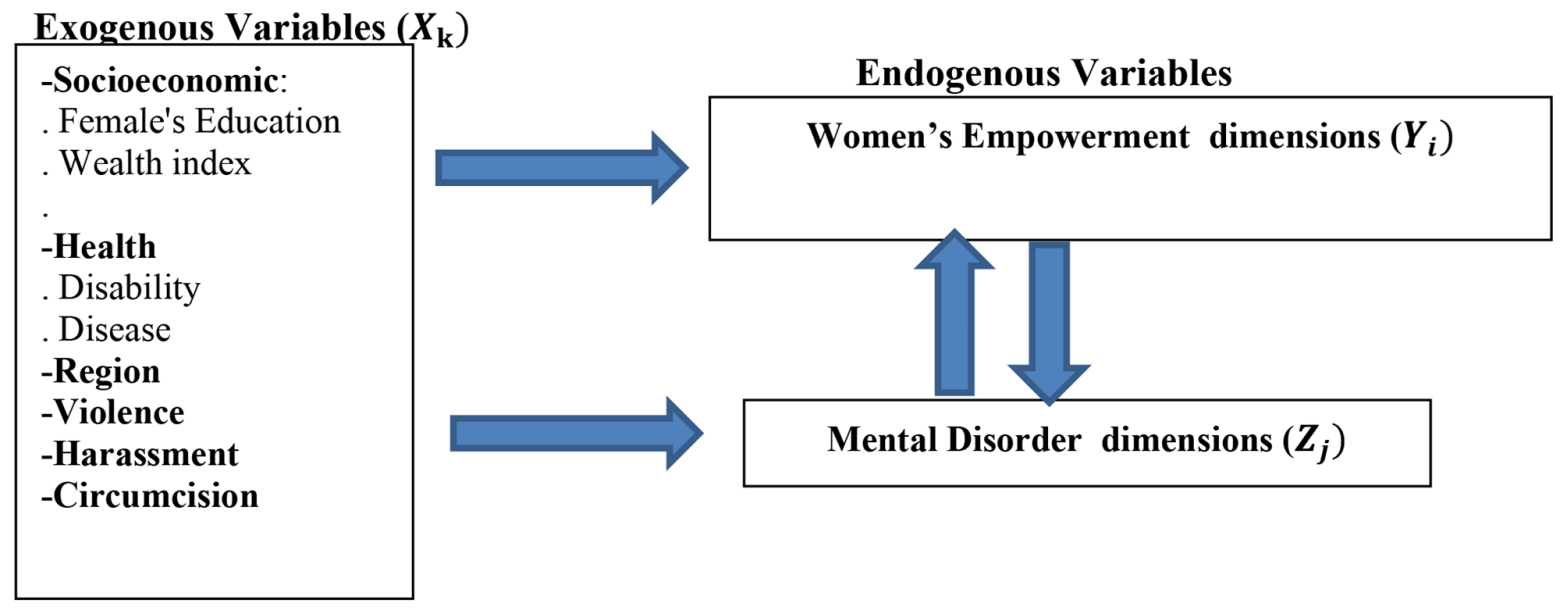
The conceptual framework for the simultaneous relationship between women’s empowerment and mental disorder

**According to our readings and understanding of the relationship between women’s empowerment and mental disorder, we conceptualize the simultaneous relationship between the two variables using the following conceptual framework after controlling for the women’s background characteristics (See** Fig. 1**).**

Consequently, the main objective of this paper is to examine whether there is a simultaneous relationship between women’s empowerment and mental disorder using SYPE 2014 as a cross sectional data. The study will depend on structural equation models to answer four questions:

1. To what extent do women’s empowerment affect women’s mental disorder and vice versa?
2. Is the relationship between women’s empowerment and mental disorder a two-way simultaneous relationship?
3. What are the significant dimensions of women’s empowerment and mental disorder that affect each other simultaneously?
4. What are the most important determinants affecting women’s empowerment and mental disorder?

## Data and methodology

### Data

The study depends on the data from the “Survey of Young People in Egypt” (SYPE) 2014. This survey was conducted by the Population Council in Egypt and disseminated by the Economic research forum. in 2014^1^. This dataset contains a module on mental health in Egypt. The SYPE included 20 WHO-validated questions to measure overall mental health status. Each person is asked to respond yes or no to a series of 20 questions about experiencing different physical symptoms of psychiatric disturbance, feelings of nervousness, uncertainty, interest in daily activities, and sense of self-worth. The survey offers information on mental health, schooling, empowerment, employment, earnings and many other topics that have deep implications for government policies for Egyptian youth. The data included 5828 women aged 14–35 years. Only married women aged 14–35 are included in the analysis, since the women empowerment index is calculated based on many indicators representing the relationship between the woman and her husband. As a result, 3052 women were used to achieve the objectives of the study.

### Methodology

#### Explanatory and confirmatory factor analysis

The recursive and nonrecursive structural equation models are mainly based on two latent constructs, namely, women’s empowerment and women’s mental disorder. The study measures mental disorder depending on a specific module within SYPE 2014. This module consists of twenty questions measuring mental disorder. On the other hand, the study constructs a women empowerment index using 10 questions based on previous literature. The exploratory factor analysis (EFA) technique is used to determine the dimensionality of items measuring every construct—whether all items fall into one dimension or fall into several dimensions [25, 26]. The EFA shows that every latent construct consists of few dimensions, where each dimension has a certain number of items.

##### Dimensions of women empowerment

Based on the above EFA technique, three dimensions are extracted for the measurement of women’s empowerment; the results show that the first dimension includes five items that explain women’s empowerment in discussing certain issues with her spouse and is titled “discussion with spouse”. The second dimension includes three items representing the “relationship with spouse”. The third dimension involves two items and represents the “treatment by spouse” (See Table 1).

**Table 1:** The extracted dimensions of female empowerment

Dimension 1	Dimension 2	Dimension 3
Discussion with spouse	Relationship with spouse	Treatment by spouse
How often does the woman discuss with her spouse? A) Her plans for the future	To what extent the woman agrees that she has enough freedom & independence in her relationship with her spouse	To what extent the woman agrees that her spouse respect her
B) Problems in her work/school	Woman and her spouse understand each other well	To what extent the woman agrees that her spouse does not treat her harshly
C) About her daily life	To what extent the woman agrees that when they have a disagreement, they talk to reach a solution	
D) Children’s future		
E) Her marital sexual relations		

Source: Calculated by authors using “Survey of Young People in Egypt” (SYPE) 2014. The Population Council in Egypt

##### Dimensions of mental disorder

EFA extracted five dimensions for women’s mental disorder index. The first dimension that represents women’s mental disorders includes six items, namely, “difficulty in making decision and enjoying life”. The second dimension involves four items; it represents “bad sleep and easily frightened”. The third dimension includes three items and represents “general fatigue’’. The fourth dimension includes three items and represents “frustrated”. The last dimension includes three items and represents “physical complications” (See Table 2).

**Table 2:** The extracted factors of female mental disorder

Dimensions	Questions (Items)
**Factor 1: Difficulty in making decisions and enjoying life**	Does the woman feel nervous, tense or worried?
	Does the woman have trouble thinking clearly?
	Does the woman feel unhappy?
	Does the woman cry more than usual?
	Does the woman find it difficult to enjoy her daily activities?
	Does the woman find it difficult to make decisions?
**Factor 2: Bad sleep and easily frightened**	Does the woman often have headaches?
	Is her appetite poor?
	Does she sleep badly?
	Is she easily frightened?
**Factor 3: General fatigue**	Is she unable to resume her daily work?
	Does the woman feel tired all the time?
	Is she easily tired?
**Factor 4: Frustrated and disappointed**	Does the woman lose interest in things?
	Does she feel that she is a worthless person?
	Has the thought of committing suicide been on her mind?
**Factor 5: Physical complications**	Is her digestion poor?
	Does her hand shake?
	Does the woman have uncomfortable feelings in her stomach?

Source: Calculated by authors using “Survey of Young People in Egypt” (SYPE) 2014. The Population Council in Egypt

Additionally, the study depends on eight exogenous observed variables which are women’s education, place of residence, exposure to violence, circumcision, sexual harassment, wealth index and health represented by two variables: having any disease and disability.

The study uses confirmatory factor analysis (CFA) to validate the measurement model. The measurement model specifies the relationship between a latent construct and one or more items in terms of factor loadings and errors of measurement [27]. The study performed CFA for all latent constructs involved in the study before modelling their interrelationships in a structural equation model (SEM). The CFA method can assess the unidimensionality, validity and reliability of a latent construct [28]. Unidimensionality is achieved when all measuring items of the latent construct have acceptable factor loadings (exceeding 0.5), while validity is the ability of the instrument to measure what is supposed to be measured for a latent construct. Additionally, reliability is measured by whether Cronbach’s alpha exceeds 0.7, composite reliability $\ge$0.6 or average variance extracted$\ge$0.5. Once the above requirements have been met, the study can proceed into structural equation modelling (SEM) [29].

#### Structural equation models

Structural equation models (SEMs) are multiple-equation regression models. SEM enables testing a set of regression equations simultaneously. SEM software can test traditional models, but it also permits the examination of more complex relationships and models, such as confirmatory factor analysis and time series analyses [30]. AMOS, which stands for analysis of moment structures, is a statistical software that is added to the SPSS module^2^, and it is specifically used for structural equation modelling. AMOS depends on a two-step model building approach that highlights the analysis of two conceptually distinct sub-models: a measurement model and a structural model [31–33].

The main objective of the study is to examine whether the relationship between women’s empowerment dimensions and mental disorder dimensions is a one-way or two-way simultaneous relationship. The study depends on two types of structural equation models, recursive and nonrecursive models, to achieve this goal.

##### Recursive structural equation model

Recursive structural equation models permit only unidirectional effects that are causality flows in only one direction. These models do not include any reciprocal causation or feedback loops [34]. The recursive structural equation model is used to test whether there is a one-way relationship between dimensions of women’s empowerment and dimensions of mental disorder.

The recursive structural equation model of women’s empowerment dimensions on mental disorder dimensions can be expressed in the following equations:

$${Y}_{i}={\lambda }_{ik}{X}_{k}+{\nu }_{i}$$

1 $${Z}_{j} ={\beta }_{ji} {Y}_{i}+ {{\sigma }_{jk}X}_{k} +{u}_{j},$$

where i = 1,2,3 (${\text{Y}}_{i}$: women’s empowerment dimensions); j = 1,2,3,4,5 (${Z}_{j}$: mental disorder dimensions); and k = 1,2,3,4,5,6,7,8 (${X}_{\text{k}}$: exogenous observed variables).

The recursive structural model of women’s mental disorder dimensions on women’s empowerment dimensions can be expressed in the following equations:

$${Z}_{j}={\alpha }_{jk}{X}_{k}+{\nu }_{j}$$

2 $${{\text{Y}}_i}{\text{ = }}{\beta _{ij}}{Z_j} + {\sigma _{ik}}{X_k}{\text{ + }}{\mu _i}$$

where i = 1,2,3; j = 1,2,3,4,5; and k = 1,2,3,4,5,6,7,8.

##### Nonrecursive structural equation model

Nonrecursive structural equation models deal with reciprocal causation and feedback loops [35, 36]. The study uses a nonrecursive structural equation model to examine whether women’s empowerment and mental disorder affect each other simultaneously.

The nonrecursive structural equation model for women’s empowerment and mental disorder through their dimensions can be expressed in the following equations:

$${Y}_{i}= {\beta }_{ij}{Z}_{j} + {{\sigma }_{ik}X}_{k} +{u}_{i}$$

3 $${{\text{Z}}_j}{\text{ = }}{\beta _{ji}}{Y_j} + {\sigma _{jk}}{X_k}{\text{ + }}{\mu _j}$$

where i = 1,2,3; j = 1,2,3,4,5; and k = 1,2,3,4,5,6,7,8,

and where ${Y}_{i}$ and${Z}_{j}$ are the endogenous latent constructs, X’s are the exogenous observed variables and${u}_{\text{i}}{, u}_{\text{j}}$ are error terms.

## Results

The results section is divided into three parts in accordance to the objectives of the study. The results start with studying the impact of women empowerment dimensions on mental disorder dimensions. This is followed with investigating the impact of mental disorder dimensions on women’s empowerment dimensions. Finally, we explore the simultaneous relationship between women’s empowerment dimensions and mental disorder dimensions. As mentioned above in the methodology, the study uses confirmatory factor analysis to validate the measurement model and demonstrates the procedure (pooled CFA) for assessing the measurement model of latent constructs. Accordingly, the Cronbach’s alpha, CR, and AVE for every dimension and all model-fit indices achieved the level of acceptance for the measurement model and the study can proceed to the structural models (see Table 3).

**Table 3:** Measurement model fit indices

Name of category	Name of index	Full name of index	Value of index in the model	References	Level of acceptance
1-Absolute Fit	RMSEA	Root Mean Square of Error Approximation	0.033	Steiger and Lind [37]	RMSEA < 0.08
2-Incremental Fit	AGFI	Adjusted Goodness of Fit	0.946	Joreskog and Sorbom [38]	AGFI > 0.9
	CFI	Comparative Fit Index	0.96	Bentler [39]	CFI > 0.9
	TLI	Tucker–Lewis Index	0.903	Tucker and Lewis [40]	TLI > 0.9
	NFI	Normed Fit Index	0.902	Bentler and Bonett [41]	NFI > 0.9
3-Parsimonious Fit	CMIN	Chisq\d.f.	4.2	Joreskog [42]	CMIN < 5

Source: Calculated by authors using “Survey of Young People in Egypt” (SYPE) 2014

### Impact of women’s empowerment dimensions on women’s mental disorder dimensions

The study attempts to answer the first research question about the impact of women’s empowerment on their mental disorder through their dimensions, controlling for the eight observed exogenous variables, where the dimensions of women’s empowerment act as mediating variables (see Table 4). Table 4 shows the one-way recursive structural model of the impact of women’s empowerment dimensions on women’s mental disorder dimensions. The results of the table are classified into two parts. The first part shows the main determinants of women’s empowerment dimensions, where in this model, the exogenous variables directly affect women’s empowerment dimensions. The model shows that women’s education (*p*-value < 0.001), areas (*p*-value < 0.05), exposure to sexual harassment (*p*-value < 0.05) and violence (*p*-value < 0.05) are the most significant determinants of women’s empowerment dimensions. When education of women increases to higher education, the discussion of women with their spouses increases by 5% and the relationship with their spouses increases by 11% compared with those with low education level.

**Table 4:** Maximum likelihood estimates of the recursive structural model of the impact of female empowerment dimensions on female mental disorder dimensions

Endogenous latent variables		Exogenous variables	Estimate	Standard error	Critical ratio
**Main direct determinants of women’s empowerment dimensions**					
Discussion with spouse	<---	Education	0.05***	0.011	4.479
	<---	Exposure to violence	-0.024*	0.011	-2.245
	<---	Wealth index	0.151***	0.039	3.851
	<---	Areas (rural-reference)	0.023*	0.012	2.021
	<---	Exposure to sexual harassment	0.096***	0.012	8.287
	<---	Woman circumcised	-0.037	0.019	-1.909
	<---	Disability	-0.081	0.07	-1.15
	<---	Disease	0.015	0.011	1.411
Relationship with spouse	<---	Education	0.029***	0.007	4.125
	<---	Exposure to violence	-0.007	0.007	-1.065
	<---	Wealth index	0.04	0.024	1.659
	<---	Areas (rural-reference)	0.015*	0.007	2.129
	<---	Exposure to sexual harassment	0.004	0.007	0.619
	<---	Woman circumcised	0.005	0.012	0.415
	<---	Disability	-0.071	0.043	-1.628
	<---	Disease	0.01	0.007	1.506
Treatment by spouse	<---	Education	0.11***	0.017	6.394
	<---	Exposure to violence	-0.045**	0.016	-2.811
	<---	Wealth index	0.05	0.059	0.849
	<---	Areas (rural-reference)	-0.143***	0.018	-7.84
	<---	Exposure to sexual harassment	-0.034*	0.017	-1.988
	<---	Woman circumcised	0.044	0.029	1.498
	<---	Disability	-0.158	0.107	-1.48
	<---	Disease	-0.027	0.017	-1.627
**Factors affecting mental health dimensions with the occurrence of women’s empowerment mediating variables**					
Difficulty in thinking	<---	Education	0.132***	0.027	4.908
	<---	Exposure to violence	-0.019	0.022	-0.868
	<---	Wealth index	0.022	0.08	0.277
	<---	Areas (rural-reference)	-0.169***	0.03	-5.561
	<---	Exposure to sexual harassment	-0.025	0.023	-1.064
	<---	Woman circumcised	0.07	0.04	1.749
	<---	Disability	-0.148	0.145	-1.015
	<---	Disease	0.023	0.023	0.993
General fatigue	<---	Education	0.192***	0.037	5.145
	<---	Exposure to violence	-0.045	0.031	-1.435
	<---	Wealth index	-0.004	0.111	-0.038
	<---	Areas (rural-reference)	-0.211***	0.042	-5.034
	<---	Exposure to sexual harassment	-0.013	0.033	-0.383
	<---	Woman circumcised	0.075	0.056	1.351
	<---	Disability	-0.174	0.202	-0.862
	<---	Disease	0.051	0.032	1.625
Frustrated	<---	Education	0.047***	0.011	4.382
	<---	Exposure to violence	0.004	0.009	0.478
	<---	Wealth index	0.027	0.031	0.873
	<---	Areas (rural-reference)	-0.069***	0.012	-5.654
	<---	Exposure to sexual harassment	-0.008	0.009	-0.814
	<---	Woman circumcised	0.026	0.016	1.661
	<---	Disability	-0.018	0.057	-0.309
	<---	Disease	0.019*	0.009	2.172
Bad in sleep	<---	Education	0.167***	0.036	4.686
	<---	Exposure to violence	-0.031	0.03	-1.028
	<---	Wealth index	-0.003	0.106	-0.024
	<---	Areas (rural-reference)	-0.194***	0.04	-4.846
	<---	Exposure to sexual harassment	-0.011	0.031	-0.35
	<---	Woman circumcised	0.114*	0.053	2.147
	<---	Disability	-0.145	0.193	-0.753
	<---	Disease	0.1***	0.03	3.329
Physical complications	<---	Education	0.128***	0.023	5.455
	<---	Exposure to violence	0.007	0.02	0.36
	<---	Wealth index	0.02	0.07	0.284
	<---	Areas (rural-reference)	-0.109***	0.026	-4.152
	<---	Exposure to sexual harassment	-0.019	0.02	-0.912
	<---	Woman circumcised	0.05	0.035	1.434
	<---	Disability	-0.079	0.126	-0.627
	<---	Disease	0.05*	0.02	2.544
***Impact of women’s empowerment dimensions on women’s mental disorder dimensions***					
Difficulty in thinking	<---	Discussion with spouse	0.024	0.013	1.881
	<---	Relationship with spouse	-0.166***	0.03	-5.618
	<---	Treatment by spouse	-1.362***	0.141	-9.696
General fatigue	<---	Discussion with spouse	0.012	0.022	0.562
	<---	Relationship with spouse	-0.276***	0.049	-5.61
	<---	Treatment by spouse	-1.862***	0.191	-9.738
Frustrated	<---	Discussion with spouse	-0.005	0.007	-0.729
	<---	Relationship with spouse	-0.097***	0.017	-5.579
	<---	Treatment by spouse	-0.51***	0.057	-8.919
Bad in sleep	<---	Discussion with spouse	0.059**	0.023	2.621
	<---	Relationship with spouse	-0.173***	0.05	-3.452
	<---	Treatment by spouse	-1.753***	0.184	-9.541
Physical complications	<---	Discussion with spouse	0.013	0.018	0.746
	<---	Relationship with spouse	-0.125**	0.039	-3.209
	<---	Treatment by spouse	-1.113***	0.12	-9.293

Source: Calculated by authors using “Survey of Young People in Egypt” (SYPE) 2014

Note that *0.01 < *p* < .05, **0.001 < *p* < .01, and ***p < .001. Two- tailed tests

On the other hand, exposure to violence decreases the discussion of women with their husbands by 2.4% compared with those who did not expose to violence. Additionally, exposure to violence decreases the agreement of women that their husbands respect their wives by almost 5%. Women who live in urban areas have discussion and stronger relationship with their husbands more than those who live in rural areas (*p*-value < 0.05). The wealth index has a significant impact (p-value < 0.001) on the discussion dimension between the woman and her husband, where increase in wealth increases the discussion between spouses.

The second part of the model shows that the greater the satisfaction of women’s empowerment dimensions is, the better women’s mental health dimensions and the lesser the mental disorder dimensions. Discussion with spouse does not significantly affect the mental disorder dimensions. On the other hand, the treatment and relationship with spouse dimensions are the most common dimensions that affect the mental disorder dimensions (*p*-value < 0.001) In other words, woman who has enough freedom and an understanding of her husband, who respects her and treats her well, is more likely to be psychologically stable and are less likely to be mentally disordered.

### Impact of women’s mental disorder dimensions on women’s empowerment dimensions

The first part of Table 5 shows the main determinants of the mental disorder dimensions, where they act as mediating variables in this model. The second part of the table shows the impact of mental disorder dimensions on women’s empowerment. The table shows that women’s education and wealth index have negative significant impacts on women’s mental disorder dimensions (*p*-value < 0.001, *p*-value,0.05 respectively). Higher education decreases the likelihood of women suffering from difficult thinking by almost 2% compared with those with lower education. Violence, sexual harassment, disease and disability have positive significant impacts on women’s mental disorder dimensions (p-value < 0.05). Women who suffer from any disease are more likely to suffer from general fatigue and physical complications by 7% and 10% respectively than those without diseases. Moreover, women who exposed to violence are more likely to suffer from all mental disorder dimensions by almost 4% compared to those who did not expose to violence. Additionally, women who live in urban areas have a higher likelihood of sleeping bad and suffer from physical complications by 6% and 5% respectively than those who live in rural areas. Finally, circumcision has no significant impact on either women’s mental disorder or women’s empowerment dimensions.

**Table 5:** Maximum likelihood estimates of the recursive structural model of the impact of women’s mental disorder dimensions on women’s empowerment dimensions

Endogenous latent variables		Exogenous variables	Estimate	Standard error		Critical ratio
**Main direct determinants of mental health dimensions**						
Difficulty in thinking	<---	Education	-0.021***	0.006	-3.378	
	<---	Exposure to violence	0.043***	0.006	7.092	
	<---	Wealth index	-0.05*	0.022	-2.263	
	<---	Areas (rural-reference)	0.024***	0.007	3.676	
	<---	Exposure to sexual harassment	0.023***	0.006	3.614	
	<---	Woman circumcised	0.008	0.011	0.77	
	<---	Disability	0.079*	0.04	1.972	
	<---	Disease	0.059***	0.006	9.188	
General fatigue	<---	Education	-0.02	0.01	-1.911	
	<---	Exposure to violence	0.041***	0.01	4.128	
	<---	Wealth index	-0.106**	0.036	-2.931	
	<---	Areas (rural-reference)	0.051***	0.011	4.678	
	<---	Exposure to sexual harassment	0.05***	0.011	4.783	
	<---	Woman circumcised	-0.008	0.018	-0.463	
	<---	Disability	0.137*	0.065	2.094	
	<---	Disease	0.098***	0.01	9.434	
Frustrated	<---	Education	-0.012***	0.003	-3.411	
	<---	Exposure to violence	0.027***	0.004	7.595	
	<---	Wealth index	-0.004	0.012	-0.31	
	<---	Areas (rural-reference)	0.002	0.004	0.63	
	<---	Exposure to sexual harassment	0.008*	0.004	2.294	
	<---	Woman circumcised	0.003	0.006	0.516	
	<---	Disability	0.067**	0.022	3.02	
	<---	Disease	0.031***	0.004	8.327	
Bad in sleep	<---	Education	-0.028*	0.011	-2.59	
	<---	Exposure to violence	0.048***	0.01	4.677	
	<---	Wealth index	-0.088*	0.038	-2.349	
	<---	Areas (rural-reference)	0.055***	0.011	4.881	
	<---	Exposure to sexual harassment	0.053***	0.011	4.886	
	<---	Woman circumcised	0.034	0.019	1.812	
	<---	Disability	0.139*	0.068	2.043	
	<---	Disease	0.146***	0.011	13.004	
Physical complications	<---	Education	0.002	0.008	0.243	
	<---	Exposure to violence	0.057***	0.008	6.948	
	<---	Wealth index	-0.038	0.029	-1.286	
	<---	Areas (rural-reference)	0.048***	0.009	5.383	
	<---	Exposure to sexual harassment	0.019*	0.009	2.288	
	<---	Woman circumcised	0	0.015	0.009	
	<---	Disability	0.1	0.053	1.883	
	<---	Disease	0.077***	0.009	9.077	
**Factors affecting women’s empowerment dimensions with the occurrence of mental health mediating variables**						
Discussion with spouse	<---	Education	0.05***	0.011	4.441	
	<---	Exposure to violence	-0.016	0.011	-1.439	
	<---	Wealth index	0.154***	0.039	3.904	
	<---	Areas (rural-reference)	0.023*	0.012	1.984	
	<---	Exposure to sexual harassment	0.097***	0.012	8.218	
	<---	Woman circumcised	-0.042*	0.019	-2.152	
	<---	Disability	-0.056	0.071	-0.784	
	<---	Disease	0.02	0.012	1.664	
Relationship with spouse	<---	Education	0.025***	0.007	3.625	
	<---	Exposure to violence	0.004	0.007	0.638	
	<---	Wealth index	0.032	0.024	1.342	
	<---	Areas (rural-reference)	0.02**	0.007	2.761	
	<---	Exposure to sexual harassment	0.01	0.007	1.395	
	<---	Woman circumcised	0.003	0.012	0.29	
	<---	Disability	-0.039	0.043	-0.916	
	<---	Disease	0.025***	0.007	3.407	
Treatment by spouse	<---	Education	0.091***	0.018	5.025	
	<---	Exposure to violence	-0.012	0.018	-0.677	
	<---	Wealth index	0.003	0.063	0.054	
	<---	Areas (rural-reference)	-0.121***	0.019	-6.297	
	<---	Exposure to sexual harassment	-0.02	0.018	-1.099	
	<---	Woman circumcised	0.063*	0.031	2.001	
	<---	Disability	-0.06	0.115	-0.523	
	<---	Disease	0.028	0.019	1.47	
***Impact of women’s mental disorder dimensions on women’s empowerment dimensions***						
**Endogenous latent variables**		**Endogenous latent variable**	**Estimate**	**Standard error**	**Critical ratio**	
Discussion with spouse	<---	Physical complications	-0.092	0.061	-1.492	
	<---	Bad in sleep	0.104*	0.041	2.518	
	<---	Frustrated	-0.49***	0.144	-3.406	
	<---	General fatigue	-0.123*	0.057	-2.148	
	<---	Difficulty in thinking	0.247*	0.11	2.239	
Relationship with spouse	<---	Physical complications	-0.048	0.037	-1.297	
	<---	Bad in sleep	0.035	0.025	1.385	
	<---	Frustrated	-0.323***	0.088	-3.677	
	<---	General fatigue	-0.1**	0.035	-2.867	
	<---	Difficulty in thinking	0.061	0.066	0.912	
Treatment by spouse	<---	Physical complications	-0.035	0.099	-0.358	
	<---	Bad in sleep	-0.215**	0.067	-3.203	
	<---	Frustrated	-0.617**	0.228	-2.703	
	<---	General fatigue	-0.097	0.092	-1.06	
	<---	Difficulty in thinking	0.039	0.176	0.222	

Source: Calculated by authors using “Survey of Young People in Egypt” (SYPE) 2014

Note that *01 < *p* < .05, **0.001 < *p* < .01, and ***p < .001. Two- tailed tests

The model also shows that the frustrated and fatigue dimensions have negative significant impacts (p-value < 0.001) on women’s empowerment dimensions. This means that a woman who loses interest, feels worthless as a person and has the idea of committing suicide on her mind is more likely to be less empowered. On the other hand, as expected, physical complications do not affect women’s empowerment.

### The two-way simultaneous relationship between women’s empowerment dimensions and mental disorder dimensions

To answer the research question that is concerned with the existence of a simultaneous relationship between women’s empowerment and their mental disorder, the nonrecursive structural equation model is applied. The results show that there is a partial two-way simultaneous relationship between women’s empowerment and mental disorder. It is called partial because not all women’s empowerment dimensions and mental disorder dimensions affect each other simultaneously, as shown in Table 6.

**Table 6:** Maximum likelihood estimates of the nonrecursive structural equation model of the simultaneous relationship between women’s empowerment and mental disorder dimensions

Endogenous latent variables		Endogenous latent variables	Estimate	Standard error	Critical ratio
General fatigue	<---	Discussion with spouse	0.387*	0.151	2.562
Discussion with spouse	<---	General fatigue	0.75*	0.351	2.137
Frustrated	<---	Discussion with spouse	0.205***	0.051	4.043
Discussion with spouse	<---	Frustrated	-2.214***	0.597	-3.708
Bad in sleep	<---	Discussion with spouse	-0.406*	0.203	-2.004
Discussion with spouse	<---	Bad in sleep	0.694**	0.215	3.226
Physical complications	<---	Discussion with spouse	0.466***	0.086	5.414
Discussion with spouse	<---	Physical complications	-0.652*	0.277	-2.356
Relationship with spouse	<---	Frustrated	4.196***	0.883	4.75
Frustrated	<---	Relationship with spouse	-0.695***	0.133	-5.232
General fatigue	<---	Treatment by spouse	-3.7***	0.551	-6.719
Treatment by spouse	<---	General fatigue	0.673*	0.285	2.364
Difficulty in thinking	<---	Relationship with spouse	1.252***	0.239	5.234
Relationship with spouse	<---	Difficulty in thinking	-2.917***	0.578	-5.042

Source: Calculated by authors using “Survey of Young People in Egypt” (SYPE) 2014

Only significant variables are shown in the table

Note that *01 < *p* < .05, **0.001 < *p* < .01, and ***p < .001. Two-tailed tests

The results of Table 6 answer the third research question, which addresses the most significant dimensions of women’s empowerment and mental disorder that affect each other simultaneously. The results show that “discussion with spouse” is the dimension (p-value < 0.05) that affects all mental disorder dimensions simultaneously. At the same time, frustrated, fatigued and frightened women with some physical complications are less likely to have discussions with their spouses (p-value < 0.001, p-value < 0.05, p-vale < 0.05 respectively). Additionally, there is a simultaneous relationship between a frustrated woman and her feelings of freedom, independence and mutual understanding with her husband (relationship with spouse), as shown in Table 6. Finally, the “treatment with spouse” dimension and “general fatigue” affect each other simultaneously.

## Discussion

The findings of the study show that women’s education plays an important positive role on women’s empowerment and mental disorder dimensions. These results are in agreement with the literature which proved hat women’s educational level is an important predictor for dimensions of women’s empowerment [12, 13]. The findings also reveal that the more the woman is educated, the better her mental health will be; this result matches the literature which showed that education reduces the transition to depression [14]. Consistent with previous studies [10], the results of this paper show that women with a high wealth index have a positive significant impact on the dimensions of women’s empowerment. Low wealth also has a significant positive impact on mental disorder dimensions, which is in agreement with the literature that showed that the odds ratio of reporting high psychological distress was greater in the lowest wealth quintile compared with the highest [15]. The study indicates that any deterioration in general health as represented by having any disease or disability has a significant positive impact on mental disorder dimensions. This result supports the declaration that states “there is no health without mental health” [3]. Our findings are also consistent with the definition of health: “not merely the absence of disease or infirmity”, but rather, “a state of complete physical, mental and social well-being” [4].

The results show that place of residence has a significant impact on women’s empowerment and mental disorder dimensions. Women who live in urban areas are more empowered but can have more mental disorders than those who live in rural areas. This is in agreement to the literature showing the significant effects of different areas on mental health [43].

Sexual harassment has a negative significant impact on treatment with spouse dimension but positive significant impact on all mental disorder dimensions (*p*-value < 0.001), which is consistent with the literature results that showed that sexual harassment was found to be a predictor of negative mental health in the forms of depression, anxiety and stress among nurses in public hospitals [44]. Additionally, exposure to violence has a negative significant impact (*p*-value < 0.05) on women’s empowerment dimensions but positive significant impact on women mental disorder dimensions. The literature states that women subjected to violence have a higher risk of mental illness, including depression and anxiety [16].

On the other hand, the findings show the one-way impact of women empowerment dimensions on mental disorder dimensions. The greater the satisfaction of women’s empowerment dimensions is, the lesser the mental disorder dimensions. The treatment and relationship with spouse dimensions are the most common dimensions that affect the mental disorder dimensions. Additionally, the findings also show the other way direction that frustrated and fatigue dimensions have negative significant impacts on women’s empowerment dimensions. This means that a woman who loses interest and feels worthless as a person is more likely to be less empowered. Finally, the results show that there is a partial two-way relationship between women’s empowerment dimensions and mental disorder dimensions, indicating that they affect each other simultaneously. The results show that “discussion with spouse” is the dimension that affects all mental disorder dimensions simultaneously. Additionally, there is a simultaneous relationship between a frustrated woman and her feelings of freedom, independence and mutual understanding with her husband (relationship with spouse). Finally, the “treatment with spouse” dimension and “general fatigue” affect each other simultaneously.

## Policy implications

Based on the results of the study, the following recommendations can be introduced:

- Emphasizing the value of woman education by state programs, especially those targeting adult education and literacy, is necessary.
- Public programs and interventions should be encouraged to alleviate poverty, especially among females. According to the results, poverty was negatively related to both women’s empowerment and mental health.
- Supporting parents to treat their female children in a good manner and avoid female violence, such as beating, hitting or shouting at them, since parents’ violence has a positive significant impact on women’s mental disorder, as depicted in our results.
- Strong penalties and strict execution of the law against sexual harassment need to be imposed to eliminate this dangerous phenomenon.
- Since health is one of the important determinants affecting female mental disorder, more government and NGO efforts to establish more rehabilitation centres for disabled women to accept themselves and their disability problems and to learn skills to help them gain economic power are highly needed.
- Encourage females to make periodic check-ups to monitor their health.
- Increase awareness of the importance of psychological counselling and treatment for woman mental disorders.
- Evaluate the role of physical, psychological and psychosocial interventions in the treatment of mental health problems.
- Emphasize the role of family counselling programs to create a healthy family in both psychological and social aspects.

## Limitations

One of the main limitations is that the study depends on 2014 survey data (SYPE 2014). However, this survey is the latest data in Egypt that includes a mental health module. Additionally, this survey does not include empowerment as a separate module to measure women’s empowerment. Thus, the study had to depend on previous research to calculate the women empowerment index while depending on the available limited variables in the SYPE data.

Finally, additional research is needed to understand the impact of mental health on women’s empowerment in Egypt after COVID-19.

## Conclusions

Regarding the one-way relationship between women’s empowerment and mental disorder, the results show that the more empowered the woman is, the better her mental health. Considering the two-way simultaneous relationship, the findings show that there is a partial two-way simultaneous relationship between women’s empowerment and mental disorder, where not all dimensions affect each other simultaneously. Women’s education has a positive impact on women’s empowerment, and the more the woman is educated, the better her mental health is. Wealth status and place of residence are the next most significant factors affecting women’s empowerment and mental disorder. Women who live in urban areas are more empowered but have a higher chance of being mentally disordered than those who live in rural areas. Sexual harassment and exposure to violence have negative impacts on women’s empowerment and mental health.

## Data Availability

For the data used in this study (SYPE 2014), the Population Council is the organization that conducted the fieldwork and collected the data, but it is disseminated by the Economic Research Forum (ERF). The dataset [SYPE 2014] used in the analysis is available in the [ERF portal] upon reasonable request at. [ https://www.erfdataportal.com/index.php/catalog/98]. The data that support the findings of this study are available from [ERF portal] but restrictions apply to the availability of these data, which were used under license for the current study, and so are not publicly available [ To get the data, all researchers-who want to have access to the data-, need to submit a proposal to the (ERF) indicating the objectives of the study, how the data will be used, and a request to use the data, then the ERF reviews the proposal and upon approval, the researcher can use the data at no cost].

## Footnotes

1 Egypt - Survey of Young People in Egypt, SYPE 2014 (erfdataportal.com).

2 SPSS: Statistical Package for the Social Sciences.

## Code availability

All program run using SPSS and AMOS version 22.
